# Human mesenchymal stromal/stem cells acquire immunostimulatory capacity upon cross-talk with natural killer cells and might improve the NK cell function of immunocompromised patients

**DOI:** 10.1186/s13287-016-0353-9

**Published:** 2016-07-07

**Authors:** Rongtao Cui, Heike Rekasi, Monika Hepner-Schefczyk, Kai Fessmann, Robert M. Petri, Kirsten Bruderek, Sven Brandau, Marcus Jäger, Stefanie B. Flohé

**Affiliations:** Department of Orthopedics and Trauma Surgery, University Hospital Essen, University of Duisburg-Essen, Virchowstraße 171, D-45147 Essen, Germany; Department of Otorhinolaryngology, University Hospital Essen, University Duisburg-Essen, Essen, Germany

**Keywords:** Mesenchymal stromal cells, Mesenchymal stem cells, Natural killer cells, Injury, Trauma, Interferon gamma, Chemokines, CCR2, CCL2, MCP-1, Immunosuppression

## Abstract

**Background:**

The suppressive effect of mesenchymal stromal/stem cells (MSCs) on diverse immune cells is well known, but it is unclear whether MSCs additionally possess immunostimulatory properties. We investigated the impact of human MSCs on the responsiveness of primary natural killer (NK) cells in terms of cytokine secretion.

**Methods:**

Human MSCs were generated from bone marrow and nasal mucosa. NK cells were isolated from peripheral blood of healthy volunteers or of immunocompromised patients after severe injury. NK cells were cultured with MSCs or with MSC-derived conditioned media in the absence or presence of IL-12 and IL-18. C-C chemokine receptor (CCR) 2, C-C chemokine ligand (CCL) 2, and the interferon (IFN)-γ receptor was blocked by specific inhibitors or antibodies. The synthesis of IFN-γ and CCL2 was determined.

**Results:**

In the absence of exogenous cytokines, trace amounts of NK cell-derived IFN-γ licensed MSCs for enhanced synthesis of CCL2. In turn, MSCs primed NK cells for increased release of IFN-γ in response to IL-12 and IL-18. Priming of NK cells by MSCs occurred in a cell–cell contact-independent manner and was impaired by inhibition of the CCR2, the receptor of CCL2, on NK cells. CD56^bright^ NK cells expressed higher levels of CCR2 and were more sensitive to CCL2-mediated priming by MSCs and by recombinant CCR2 ligands than cytotoxic CD56^dim^ NK cells. NK cells from severely injured patients were impaired in cytokine-induced IFN-γ synthesis. Co-culture with MSCs or with conditioned media from MSCs and MSC/NK cell co-cultures from healthy donors improved the IFN-γ production of the patients’ NK cells in a CCR2-dependent manner.

**Conclusions:**

A positive feedback loop driven by NK cell-derived IFN-γ and MSC-derived CCL2 increases the inflammatory response of cytokine-stimulated NK cells not only from healthy donors but also from immunocompromised patients. Therapeutic application of MSCs or their soluble factors might thus improve the NK function after severe injury.

**Electronic supplementary material:**

The online version of this article (doi:10.1186/s13287-016-0353-9) contains supplementary material, which is available to authorized users.

## Background

Mesenchymal stromal/stem cells (MSCs) are nonhematopoietic, multipotent progenitor cells that may give rise to osteoblasts, chondrocytes, and adipocytes. MSCs are defined according to their lack of the hematopoietic and endothelial markers CD45 and CD34 and their expression of the stromal markers CD105, CD90, CD29, and CD73 [1]. Because of their potential for trilineage differentiation, MSCs are considered to contribute to tissue regeneration [2]. In addition, MSCs release C-C chemokine ligand (CCL) 2 (also known as monocyte-chemoattractant protein MCP-1) that recruits monocytes/macrophages to the site of injury and, thereby, supports wound healing [3].

MSCs not only possess regenerative capacity but also display immunomodulatory properties. As part of the hematopoietic stem cell niche, MSCs are tightly located to hematopoietic stem cells and are involved in the maintenance of hematopoiesis [4]. Moreover, MSCs regulate the function of finally differentiated immune cells due to the release of regulatory mediators such as prostaglandin E (PGE) 2, interleukin (IL)-10, or transforming growth factor (TGF)-β [5]. MSCs inhibit the proliferation of T cells, the release of inflammatory cytokines by dendritic cells and macrophages, the proliferation and immunoglobulin production of B cells, and the cytotoxic activity of natural killer (NK) cells [6]. This regulatory activity of MSCs has led to the development of novel therapeutic approaches in the treatment of inflammatory disorders such as graft-versus-host disease or autoimmunity. More recently it has been shown that MSCs do not act as immunosuppressant cells by “default” but may also enhance inflammation depending on the microenvironment [7].

NK cells belong to the innate immune system and can be divided into two subpopulations depending on their expression of the adhesion molecule CD56 [8]. CD56^dim^ NK cells are involved in the defense against virus infections and tumor cells through their cytotoxic activity. In contrast, CD56^bright^ NK cells possess only poor cytotoxic activity but release high amounts of interferon (IFN)-γ upon exposure to the proinflammatory cytokines IL-12 and IL-18, and thereby contribute to effective antimicrobial immune responses [9]. From previous in*-*vitro studies it is known that MSCs suppress the tumor cell-directed cytotoxicity, proliferation, and IFN-γ secretion of NK cells mainly through the release of PGE_2_ and the activity of indoleamine 2,3-dioxygenase (IDO) [10]. In contrast to these reports, we have recently shown that MSCs enhance the release of IFN-γ from a NK cell line in the context of stimulation with IL-12 and IL-18. The stimulatory effect of MSCs on the NK cells occurs in a cell–cell contact-dependent and contact-independent manner and is associated with an increased expression of the IL-12 receptor on NK cells [11].

In the present study, we investigated the underlying mechanism of MSC-mediated NK cell stimulation. We identified a novel positive feedback loop between MSCs and primary human NK cells including the release of CCL2/MCP-1 by MSCs and IFN-γ by NK cells. The stimulatory activity of MSCs largely affected the CD56^bright^ NK cell subpopulation. Moreover, MSCs improved the function of NK cells from severely injured patients who are at enhanced risk for nosocomial infections due to the development of immunosuppression [12].

## Methods

### Patients

Bone marrow aspirate was obtained from patients who underwent total hip replacement surgery. Peripheral blood was drawn from healthy donors and from severely injured patients on day 8 after injury. The patients were at least 18 years old, suffered multiple injuries such as thorax trauma, bone fracture, hemorrhage, brain injury, or soft tissue trauma, and displayed an Injury Severity Score (ISS) > 16 (Table 1). Informed consent was obtained from volunteers and from patients or their legal representatives (in case of unconsciousness). The study was performed according to the Declaration of Helsinki and was approved by the Ethics Committee of the Medical Faculty of the University Duisburg-Essen.

**Table 1:** Characteristics of healthy donors and patients

	Controls	Patients
	(*n* = 6)	(*n* = 6)
Age (years)	32 (22–37)	25 (21–45)
Gender (male/female)	2/4	4/2
ISS	–	36 (34–43)

Age and Injury Severity Score (*ISS*) are expressed as median (25th–75th interquartile range)

### Generation of MSCs

Mononuclear cells from bone marrow aspirates were isolated by density gradient centrifugation using Leucosep tubes (Greiner Bio-One, Frickenhausen, Germany) according to the manufacturer’s protocol. After washing with Dulbecco’s Phosphate Buffer Saline (DPBS; Gibco Life Technologies, Darmstadt, Germany) the cell pellet was resuspended in “MSC-medium” composed of Dulbecco’s modified Eagle’s medium (DMEM; Gibco Life Technologies) supplemented with 10 % endotoxin-tested fetal calf serum (FCS; Biochrome, Berlin, Germany), 100 U/ml penicillin, 0.1 mg/ml streptomycin, 2 mM l-glutamax, and 1 mM sodium pyruvate (all from Sigma, Deisenhofen, Germany). One million mononuclear cells were seeded in 7 ml MSC-medium per T75 cm^2^ flask (Greiner Bio-One) and were incubated in a humidified atmosphere containing 5 % CO_2_ at 37 °C. After 24 h, half of the medium was replaced by fresh MSC-medium. Forty hours later, the nonadherent cells were removed by gentle washing with phosphate-buffered saline (PBS; Gibco Life Technologies) before addition of MSC-medium. Thereafter, the medium was replaced twice a week. When the cells reached a confluency of around 80 % they were detached using 6 ml Accutase (Gibco Life Technologies), harvested, and reseeded at 10^6^ cells per flask (T75 cm^2^) in 7 ml MSC-medium. After three passages, phenotypical characterization was performed by flow cytometry [1]. All MSCs were negative for the hematopoietic or endothelial markers CD45, CD34, and CD31 but expressed the characteristic MSC markers CD29, CD73, CD90, and CD105 (see Additional file 1: Figure S1). MSCs were used in passages 3–8 throughout the experiments. MSCs from nasal mucosa (nmMSCs) were generated as described previously [13]. MSCs from five bone marrow aspirate donors and from three nasal mucosa donors were used in this study.

### Isolation of mononuclear cells and NK cells from blood

Peripheral blood mononuclear cells (PBMC) were obtained from peripheral blood density gradient centrifugation as already described. Untouched NK cells were isolated from PBMC using a negative selection human NK cell isolation kit with AutoMACS (both from Miltenyi Biotec, Bergisch Gladbach, Germany) according to the manufacturer’s instruction. The purity of CD56^+^ NK cells was >90 % as determined by flow cytometry. NK cells were cultured in “NK cell-medium” Very Low Endotoxin medium VLE RPMI-1640 (Biochrome) supplemented with 10 % FCS, 100 U/ml penicillin, 0.1 mg/ml streptomycin, and 2 mM l-glutamax (all from Sigma). Purified NK cells were directly used for the experiments. NK cells from 27 healthy donors and from six patients were used in this study.

### Cell culture

MSCs were seeded at 1.25 × 10^4^ cells per well (24-well plate; BD Biosciences, Heidelberg, Germany) in 900 μl MSC-medium. The medium was aspirated after overnight culture and 6.25 × 10^4^ NK cells in 900 μl NK cell-medium were added. As controls, MSCs and NK cells were cultured alone in NK cell-medium. In parallel, cell-free NK cell-medium was incubated under identical conditions. In one experimental set-up, NK cells were placed in a 24-well trans-well plate (0.4 μm pore size; BD Biosciences) with MSCs on the bottom of the culture plate. After 24 h, the cultures underwent one of the following three procedures: “co-culture”, NK cell mono-cultures and MSC/NK cell co-cultures were stimulated with IL-12 (1 ng/ml; Miltenyi Biotec) and IL-18 (10 ng/ml; MBL International, Woburn, MA, USA); “detachment”, NK cells from mono-cultures and NK cells that were detached from MSCS in the co-cultures were harvested by gentle pipetting, washed, and replated in fresh wells (1 × 10^4^ NK cells/well, 96-well plate; BD Biosciences) before stimulation with IL-12 (1 ng/ml) and IL-18 (10 ng/ml); and “conditioned media”, supernatants from MSCs alone, NK cells alone, MSC/NK cell-co-cultures, and the cell-free medium were harvested and served as “conditioned media” after removal of residual cells by centrifugation. The conditioned media (150 μl) were immediately transferred to freshly isolated NK cells that were seeded at 1 × 10^4^ per well (96-well plate) in 50 μl NK cell-medium. Thereafter, the cells were stimulated with IL-12 (1 ng/ml) and IL-18 (10 ng/ml) or left unstimulated as a negative control.

Alternatively, freshly isolated NK cells (1 × 10^4^ per well; 96-well plate) were cultured in the presence or absence of recombinant human CCL2/MCP-1, CCL8, CCL7, or CCL12 (all from PeproTech, Hamburg, Germany) at a final concentration of 0.5 ng/ml for 12 h before stimulation with IL-12 (1 ng/ml) and IL-18 (5 ng/ml).

Where specified, neutralizing monoclonal antibodies against CCL2/MCP-1 (clone 2H5; BioXCell, West Lebanon, NH, USA), unspecific hamster IgG isotype control antibodies (each 10 μg/ml; BioXCell), inhibitory monoclonal antibodies against the IFN-γ receptor 1 (clone GIR208; Biotechne, Wiesbaden, Germany), unspecific mouse IgG1 isotype control antibodies (each 10 μg/ml; Biotechne), a selective antagonist of the C-C-chemokine receptor (CCR) 2 (RS102895, 5 nM; Tocris Bioscience, Bristol, UK), or dimethyl sulfoxide (DMSO; Sigma) as solvent control were added to the cultures. In general, all cultures were set up in 4–5 replicates. Cell supernatants were harvested 24 h after stimulation with IL-12 and IL-18 and were stored at –20 °C.

In a separate set of experiments, MSCs were seeded as already described and treated with different concentrations of recombinant human IFN-γ (5–100 pg/ml; Miltenyi Biotec) or with PBS as control for 12 h before supernatants were harvested.

### Quantification of CCL2/MCP-1 and IFN-γ

Quantification of human CCL2/MCP-1 and IFN-γ was performed by enzyme-linked immunosorbent assay (ELISA) according to the manufacturer’s instructions (DuoSet; Biotechne). The detection limit was 15 pg/ml. For some analyses, a specialized highly sensitive IFN-γ ELISA with a detection limit of 1.6 pg/ml was used according to the manufacturer’s instructions (NBP1-91174 Interferon gamma; Novus Biologics/Biotechne).

### Flow cytometry

PBMC were incubated with fluorescent antibodies against CD3 (fluorescein isothiocyanate-labeled, clone DaA3; Immuno Tools, Friesoythe, Germany), CD56 (allophycocyanin-labeled, clone CMSSB; eBioscience, San Diego, CA, USA), and CD192/CCR2 (phycoerythrin-labeled, clone K036C2; Biolegend, San Diego, CA, USA) for 12 min at 4 °C. Thereafter, the cells were washed with Cell Wash (BD Biosciences).

For intracellular detection of IFN-γ in NK cells, 0.5 μl/ml of Golgi-Stop (Monensin; BD Biosciences) was added during the last 5 h of culture. After surface staining using anti-CD3 and anti-CD56 antibodies as already described, the cells were fixed and permeabilized using Cytofix/Cytoperm (BD Biosciences) and antibodies against IFN-γ (phycoerythrin-labeled, clone 4S.B3; Biolegend) were added. After 20 min, the cells were washed.

Appropriate isotype controls were used for all stainings. The acquisition of data was performed using FACSCalibur (BD Biosciences). Data were analyzed using Cell Quest Pro software (BD Biosciences). NK cells were identified and gated as CD3^–^CD56^bright^ or CD3^–^CD56^dim^ cells. The expression of CCR2 and IFN-γ on/in gated NK cells was quantified according to the isotype control staining.

### Statistical analyses

Data from replicate cultures are presented as mean ± SD in bar graphs and statistical analysis was performed using one-way ANOVA followed by the Bonferroni multiple comparison test. Combined data of multiple donors are presented as scatter plots and median with interquartile range. Statistical analysis was performed using the Mann–Whitney *U* test or Wilcoxon signed-rank test as indicated. GraphPad Prism 5.0 served as the software for the analyses. *p* ≤ 0.05 was considered statistically significant.

## Results

### MSCs prime NK cells for increased IFN-γ secretion

**Fig. 1:**
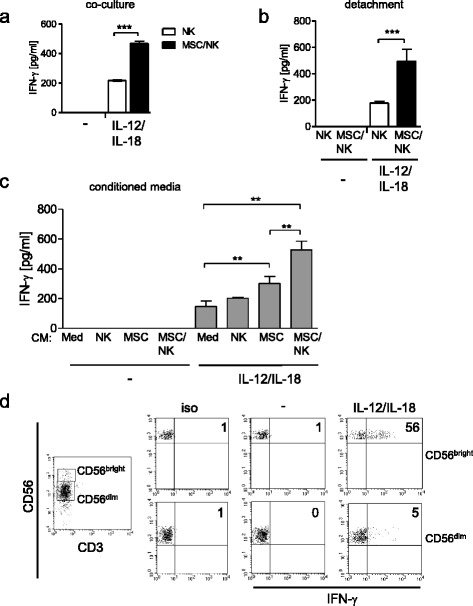
MSCs prime NK cells for increased IFN-γ release. **a**, **b** Purified NK cells from healthy volunteers were cultured in the absence (*NK*) or presence of MSCs (*MSC/NK*) for 24 h. **a** Cells were stimulated with IL-12 (1 ng/ml) and IL-18 (10 ng/ml). Unstimulated cells served as negative control. Twenty-four hours later, the content of IFN-γ in the supernatants was determined. **b** NK cells were harvested, washed, transferred into fresh plates, and stimulated or not with IL-12 and IL-18. The content of IFN-γ in the supernatants was quantified after 24 h. **c** Freshly isolated NK cells were cultured in the presence of conditioned media (*CM*) from NK cells, MSCs, MSC/NK cell co-cultures, and cell-free medium and were stimulated with IL-12 and IL-18. The release of IFN-γ into the supernatant was determined 24 h later. Bar graphs show mean ± SD of 4–5 replicates from one representative out of at least 12 independent experiments. Statistical analysis was performed using one-way ANOVA followed by Bonferroni multiple comparison test. **d** Flow cytometric analysis of intracellular IFN-γ synthesis in gated CD3^–^CD56^bright^ and CD3^–^CD56^dim^ NK cells stimulated with IL-12 and IL-18. Unstimulated cells served as negative control (–). The threshold of positive staining for IFN-γ was set according to the isotype control (*iso*). Numbers in the dot plots show the percentage of cells positive for IFN-γ. The median (interquartile range) of the percentage of IFN-γ^+^ cells among CD56^bright^ NK cells was 58.3 (55.6–67; *n* = 7). **p < 0.01, ***p < 0.001. *MSC* mesenchymal stromal/stem cell, *NK* natural killer, *IFN* interferon

We have previously shown that human bone marrow-derived MSCs prime NK-92 cells, a human NK cell line, for increased release of IFN-γ upon stimulation with IL-12 and IL-18. We investigated whether MSCs similarly modulated human primary NK cells from peripheral blood and whether the presence of MSCs during stimulation of NK cells with cytokines was required to exert their stimulatory effect on NK cells. Therefore, human primary NK cells were isolated from peripheral blood of healthy donors and were cultured alone or together with MSCs at a ratio of 5:1 (NK cells:MSCs) for 24 h before stimulation with IL-12 and IL-18. At this ratio MSCs displayed an intermediate stimulatory effect on NK cells as verified previously [11]. Unstimulated cells served as negative controls. In the absence of IL-12 and IL-18, NK cells did not release detectable levels of IFN-γ (Fig. 1a). As expected, NK cells released IFN-γ after exposure to IL-12 and IL-18. In the presence of MSCs, IFN-γ secretion by NK cells increased by 2-fold (Fig. 1a).

To address the relevance of MSCs during stimulation of NK cells, mono-cultures and co-cultures were set up. After 24 h, NK cells were carefully harvested, washed, and transferred into fresh culture plates before stimulation. NK cells that had been co-cultured with MSCs and then separated released significantly higher levels of IFN-γ than NK cells that had been cultured alone (Fig. 1b). No IFN-γ was released from reseeded NK cells in the absence of IL-12 and IL-18 (Fig. 1b).

Because contact with MSCs during stimulation of NK cells with IL-12 and IL-18 was not required to increase the release of IFN-γ, we next investigated whether soluble factor(s) derived from MSCs were responsible for the priming effect on NK cells. Therefore, “conditioned media” from NK cells alone, MSCs alone, and MSC/NK cell co-cultures were harvested and transferred to freshly isolated NK cells. Transfer of cell-free medium that had been kept under identical conditions to the cells was used as control. Thereafter, the NK cells were stimulated or left unstimulated. In the presence of conditioned medium from MSCs alone and, even more, from MSC/NK cell co-cultures, NK cells released significantly increased amounts of IFN-γ upon stimulation (Fig. 1c). The conditioned medium from NK cell mono-cultures did not change the IFN-γ production (Fig. 1c). Conditioned media did not induce the secretion of IFN-γ from NK cells in the absence of IL-12 and IL-18 (Fig. 1c).

Intracellular staining and flow cytometry revealed that CD56^bright^ NK cells were the main subpopulation that secreted IFN-γ upon stimulation (Fig. 1d). Thus, MSCs instruct primary NK cells for increased IL-12/IL-18-induced IFN-γ secretion through a soluble factor.

### MSC-derived CCL2/MCP-1 mediates NK cell priming

**Fig. 2:**
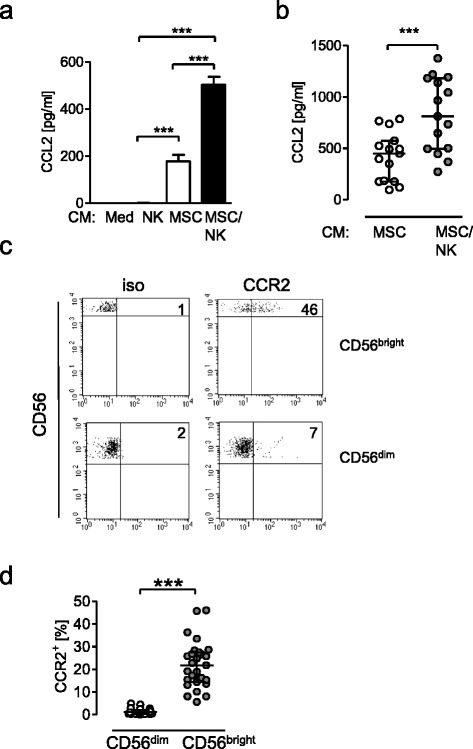
MSCs increase the release of CCL2 upon contact with NK cells and CD56^bright^ NK cells express CCR2. **a**, **b** Conditioned media (*CM*) from NK cells, MSCs, MSC/NK cell co-cultures, and cell-free medium were prepared as described in Methods and the content of CCL2 was quantified. **a** Bar graph showing mean ± SD of 4–5 replicates from one representative experiment. **b** Cumulative data of 15 experiments. No CCL2 was detectable in CM from NK cells only. Statistical analysis was performed using one-way ANOVA followed by Bonferroni multiple comparison test. **c**, **d** PBMC were stained with fluorochrome-labeled antibodies against CD3, CD56, and CCR2. **c** Representative dot plot of CCR2 expression on gated CD3^–^CD56^bright^ and CD3^–^CD56^dim^ NK cells (gated as shown in Fig. 1d). Numbers indicate the percentage of cells positive for CCR2. The threshold of positive staining for CCR2 was set according to the isotype control (*iso*). **d** Scatter plot showing the percentage of CCR2^+^ cells among CD56^bright^ and CD56^dim^ NK cells from individual healthy donors (*n* = 27). *Horizontal lines* indicate the median with interquartile range. Statistical analysis was performed using the Wilcoxon signed-rank test. ***p < 0.001. *MSC* mesenchymal stromal/stem cell, *NK* natural killer, *CCL2* C-C chemokine ligand 2, *CCR2* C-C chemokine receptor 2

We determined the content of CCL2/MCP-1 in the conditioned media from mono-cultures and co-cultures that had been prepared as already described. No CCL2/MCP-1 was detectable in the supernatant from NK cells. As expected, MSCs alone released CCL2/MCP-1 (Fig. 2a). When co-cultured with NK cells, MSCs released more CCL2/MCP-1 (Fig. 2a). The extent of CCL2 secretion varied depending on the combination of MSCs and NK cells (Fig. 2b). To evaluate whether CCL2/MCP-1 might contribute to the MSC-mediated priming of NK cells for increased IFN-γ synthesis, we first investigated whether NK cells expressed CCR2, the receptor for CCL2/MCP-1. CD56^bright^ NK cells expressed CCR2 at much higher levels than CD56^dim^ NK cells (Fig. 2c). We observed a large variation in CCR2 expression between different donors (Fig. 2d).

**Fig. 3:**
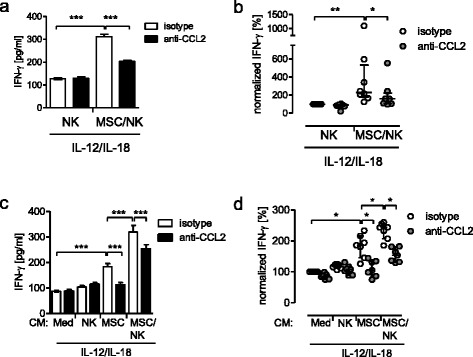
Neutralization of CCL2 diminishes the MSC-mediated stimulation of NK cells. **a**, **b** NK cells were incubated alone or in co-culture with MSCs in the presence of neutralizing antibodies against CCL2 or isotype control antibodies for 24 h. Thereafter, the cells were stimulated with IL-12 and IL-18 for 24 h and the concentration of IFN-γ in the supernatants was determined. **b** Freshly isolated NK cells were incubated with conditioned media (*CM*) from NK cells, MSCs, MSC/NK cell co-cultures, and cell-free medium each in the presence of neutralizing antibodies against CCL2 or the isotype control. Thereafter, all cells were stimulated with IL-12 and IL-18 for 24 h. The amount of IFN-γ in the supernatant was quantified. **a**, **c** Bar graphs showing mean ± SD of 4–5 replicates of one representative experiment. Statistical analysis was performed using one-way ANOVA followed by Bonferroni multiple comparison test. **b**, **d** Cumulative and normalized data of eight independent experiments. One hundred percent was equivalent to median (interquartile range) 132 (70–340) pg/ml and 87 (38–308) pg/ml in (**b**) and (**d**), respectively. Statistical analysis was performed using the Wilcoxon signed-rank test. *p < 0.05**p < 0.01***p < 0.001. *MSC* mesenchymal stromal/stem cell, *NK* natural killer, *CCL2* C-C ligand 2, *IFN* interferon

Next, we investigated the priming of NK cells by MSCs in the presence of neutralizing antibodies against CCL2/MCP-1. Mono-cultures and co-cultures were therefore set up in the presence or absence of neutralizing antibodies against CCL2/MCP-1 before stimulation. Neutralization of CCL2/MCP-1 reduced the secretion of IFN-γ in MSC/NK cell co-cultures but not in cultures of NK cells alone (Fig. 3a, b). In the absence of IL-12 and IL-18, NK cells did not secrete detectable levels of IFN-γ (data not shown).

We asked whether CCL2/MCP-1 likewise contributed to the stimulatory activity of the conditioned media when transferred to fresh NK cells. Neutralization of CCL2/MCP-1 completely and partially abrogated the stimulatory effect of the conditioned media from MSCs and from MSC/NK cell co-cultures, respectively (Fig. 3c, d).

**Fig. 4:**
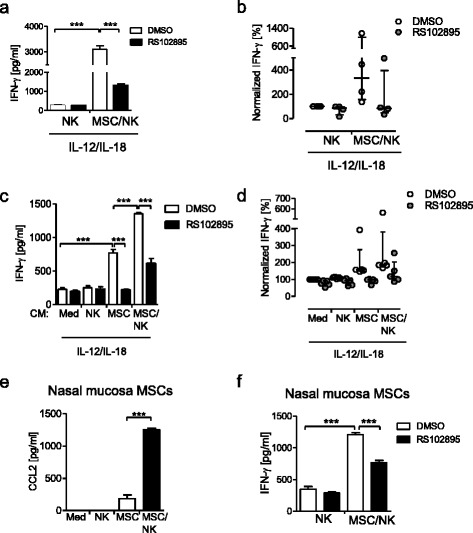
Inhibition of CCR2 attenuates the MSC-mediated activation of NK cells. **a**, **b** NK cells were treated with the CCR2 inhibitor RS102895 or with the solvent DMSO as control before they were cultured alone or in co-culture with MSCs for 24 h. Thereafter, the cells were stimulated with IL-12 and IL-18 and the supernatant was harvested after 24 h for quantification of IFN-γ. **c**, **d** Freshly isolated NK cells were treated with the CCR2 inhibitor RS102895 or with DMSO as control before addition of conditioned media (*CM*) from NK cells, MSCs, MSC/NK cell co-cultures, and cell-free medium. Thereafter, all cells were stimulated with IL-12 and IL-18 and the amount of IFN-γ in the supernatants was quantified. **a**, **c** Bar graphs showing mean ± SD of 4–5 replicates of one representative experiment. **b**, **d** Cumulative and normalized data of 4–5 independent experiments. One hundred percent was equivalent to median (interquartile range) 352 (285–386) pg/ml and 723 (371–816) pg/ml in (**b**) and (**d**), respectively. **e** CCL2 content in conditioned media from mono-cultures and co-cultures using MSCs from human nasal mucosa. **f** IFN-γ release from NK cells cultured alone or together with MSCs from human nasal mucosa each in the presence or absence of RS102895. Bar graphs showing mean ± SD of 4–5 replicates of one representative out of three independent experiments. Statistical analysis was performed using one-way ANOVA followed by Bonferroni multiple comparison test. ***p < 0.001. *CCL2* C-C ligand 2, *DMSO* dimethyl sulfoxide, *MSC* mesenchymal stromal/stem cell, *NK* natural killer, *IFN* Interferon

Likewise, inhibition of CCR2, the receptor for CCL2, with the specific inhibitor RS102895 clearly decreased the release of IFN-γ from NK cells when co-cultured together with MSCs but not when cultured alone (Fig. 4a, b). Moreover, inhibition of CCR2 completely reversed the stimulatory effect of the conditioned medium from MSCs on the NK cell-derived IFN-γ production and clearly decreased the priming that was mediated by conditioned medium from MSC/NK cell co-cultures (Fig. 4c, d). In the absence of IL-12 and IL-18, NK cells did not secrete detectable levels of IFN-γ (data not shown).

In order to investigate whether the NK cell-stimulatory activity was specific for bone marrow-derived MSCs we additionally used nmMSCs in the same experimental set-up. Upon contact with NK cells, nmMSCs strongly increased their release of CCL2 (Fig. 4e). In turn, the contact with nmMSCs stimulated NK cells for enhanced IL-12/IL-18-induced secretion of IFN-γ in a CCR2-dependent manner (Fig. 4f).

**Fig. 5:**
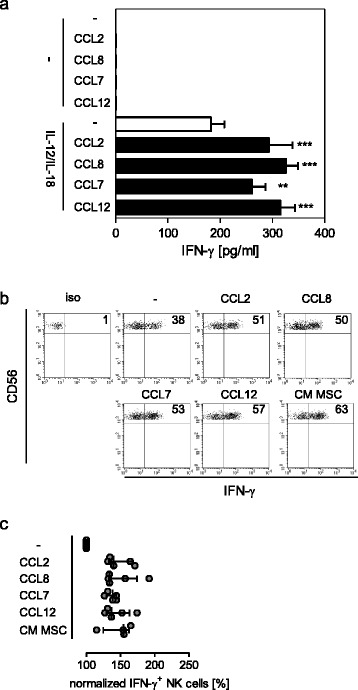
Monocyte chemotactic proteins promote IFN-γ production in NK cells. NK cells were incubated in the absence (–) or presence of recombinant human CCL2, CCL8, CCL7, and CCL12, or with conditioned medium (*CM*) from MSCs for 12 h. Thereafter, the cells were stimulated with suboptimal concentrations of IL-12 (1 ng/ml) and IL-18 (5 ng/ml) or left unstimulated. **a** After 24 h the content of IFN-γ in the supernatant was determined. Bar graph showing mean ± SD of 4–5 replicates of one representative out of five independent experiments. Statistical analysis was performed using one-way ANOVA followed by Bonferroni multiple comparison test. **b** Dot plots of intracellular staining of IFN-γ in gated CD3^–^CD56^bright^ NK cell (gating strategy as shown in Fig. 1d) after stimulation with IL-12 and IL-18. The threshold of positive staining for IFN-γ was set according to the isotype control (*iso*). Numbers indicate the percentage of IFN-γ-positive NK cells. **c** Cumulative data on the percentage of IFN-γ^+^CD56^bright^ NK cells from five donors after normalization to the percentage of cells stimulated with IL-12/IL-18 in the absence of CCL (set as 100 %). *Vertical lines* indicate the median and interquartile range. ***p < 0.001, **p < 0.01. *CCL* C-C chemokine ligand, *IFN* interferon, *MSC* mesenchymal stromal/stem cell

Having established that CCR2 and its ligand CCL2/MCP-1 contributed to the MSC-mediated priming of NK cells, we asked whether targeting CCR2 was a general mechanism that stimulates NK cells for enhanced release of IFN-γ. In addition to CCL2/MCP-1 there exist several ligands of CCR2, namely CCL8/MCP-2, CCL7/MCP-3, and CCL12/MCP-5. NK cells were therefore cultured in the presence or absence of diverse CCR2 ligands before stimulation at suboptimal levels in order to detect the synergism between the recombinant chemokines and IL-12/IL-18. As control, NK cells were treated with the CCR2 ligands alone. All ligands increased the IL-12/IL-18-induced release of IFN-γ (Fig. 5a). None of the ligands induced the release of IFN-γ in the absence of IL-12 and IL-18 (Fig. 5a). Intracellular staining revealed that the CCR2 ligands similar to conditioned medium from MSCs increased the percentage of IFN-γ-producing CD56^bright^ NK cells upon exposure to IL-12 and IL-18 (Fig. 5b, c). In contrast, CD56^dim^ NK cells did not respond to the CCR2 ligands or to the conditioned medium (Additional file 2: Figure S2). Thus, CCL2/MCP-1 is released by MSCs and MSC/NK cell co-cultures and stimulates CD56^bright^ NK cells for increased IL-12/IL-18-induced IFN-γ synthesis in a CCR2-dependent manner.

### NK cell-derived IFN-γ licenses MSCs for increased CCL2/MCP-1 release

**Fig. 6:**
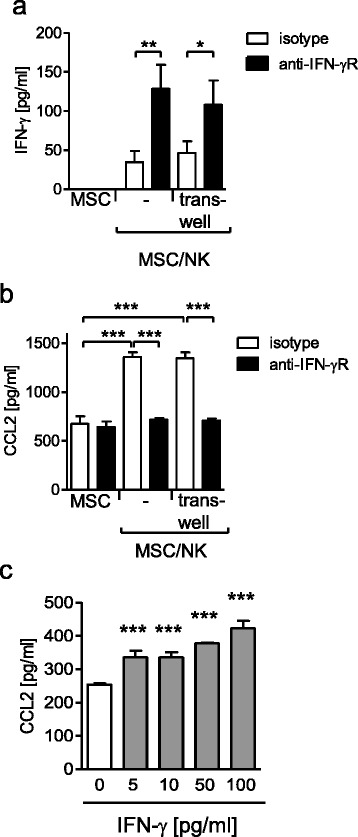
NK cell-derived IFN-γ enhances the CCL2 production from MSCs. **a**, **b** MSCs were treated with blocking antibodies against the receptor for IFN-γ (*IFN-γR*) or with isotype control antibodies. NK cells were added directly to the MSCs or were separated by a trans-well. After 24 h, the concentration of IFN-γ (**a**) and CCL2 (**b**) in the supernatants was quantified. **c** MSCs were cultured with various concentrations of recombinant human IFN-γ for 12 h and the production of CCL2 was measured. Bar graphs showing mean ± SD of 3–5 replicates from one representative out of three experiments. Statistical analysis was performed using one-way ANOVA followed by Bonferroni multiple comparison test. ***p < 0.001, **p < 0.01, *p < 0.05. *MSC* mesenchymal stromal/stem cell, *NK* natural killer, *CCL2* C-C ligand 2, *IFN* interferon

Considering that MSCs released enhanced amounts of CCL2/MCP-1 when cultured in the presence of NK cells under neutral conditions (Fig. 2a) we asked whether NK cells in turn stimulated MSCs through IFN-γ. Therefore, mono-cultures and co-cultures were set up in the presence of antibodies that blocked the IFN-γ receptor. No further cytokines were added. Moreover, co-cultures were set up in a trans-well system that allows the exchange of soluble factors but prevents direct cell-to-cell contact. The content of IFN-γ was determined using a highly sensitive ELISA. No IFN-γ was detected in supernatants from MSCs alone (Fig. 6a). IFN-γ was present at very low levels in MSC/NK cell co-cultures independent of the cell contact (Fig. 6a). Blocking of the IFN-γ receptor led to the accumulation of IFN-γ in all MSC/NK cell co-cultures but not in MSC mono-cultures (Fig. 6a). Quantification of CCL2/MCP-1 in the same supernatants revealed that the constitutive release of CCL2/MCP-1 by MSCs was not affected by blocking the IFN-γ receptor. In contrast, the increased synthesis of CCL2/MCP-1 in the MSC/NK cell co-cultures was completely abrogated in the presence of the anti-IFN-γ receptor antibodies (Fig. 6b). The separation of MSCs and NK cells during culture did not change the NK cell-mediated increase of CCL2/MCP-1 production or the suppressive effect of the anti-IFN-γ receptor antibodies on the synthesis of CCL2/MCP-1 (Fig. 6b).

To confirm that such a low amount of IFN-γ as found in the MSC/NK cell co-cultures was able to increase the release of MSC-derived CCL2/MCP-1, MSCs were cultured with low concentrations of recombinant IFN-γ. We observed that as little as 5 pg/ml IFN-γ was sufficient to significantly increase the release of CCL2/MCP-1 by MSCs (Fig. 6c). Thus, under neutral conditions NK cell-derived IFN-γ licenses MSCs for increased CCL2/MCP-1 production.

### MSC-derived CCL2/MCP-1 improves the function of NK cells of immunocompromised patients

**Fig. 7:**
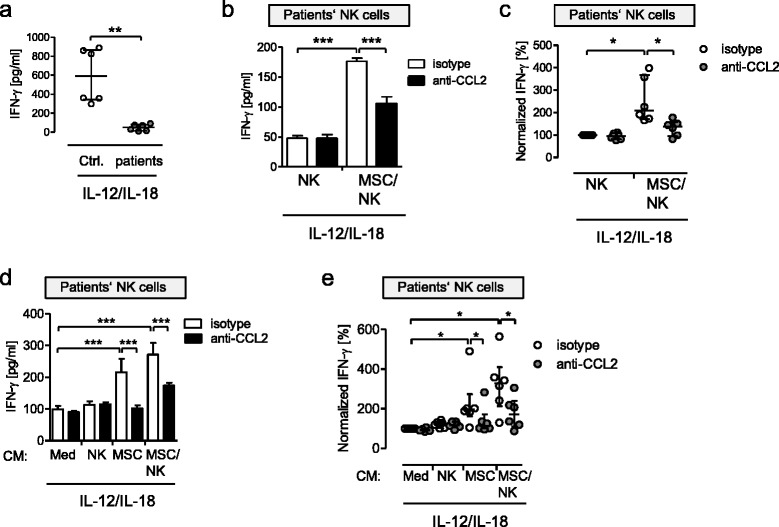
NK cells from severely injured patients exhibit impaired IFN-γ production, which is partially rescued by MSC-derived CCL2. **a** Purified NK cells from healthy donors and severely injured patients (*n* = 6) were stimulated with IL-12 and IL-18 for 24 h and the concentration of IFN-γ in the supernatants was determined. *Horizontal lines* indicate the median with interquartile range. **b**, **c** MSCs were treated with neutralizing antibodies against CCL2 or with the isotype control. Purified NK cells from patients were added to MSCs from healthy donors or were cultured alone. All cells were stimulated with IL-12 and IL-18 for 24 h and the concentration of IFN-γ in the supernatants was assessed. **b** Bar graph showing mean ± SD of 5 replicates from one representative experiment. **c** Scatter plot showing the individual values obtained from cells of six patients. Data were normalized to the values from NK cells alone stimulated in the presence of the isotype control (set as 100 %). *Horizontal lines* indicate the median with interquartile range. **d**, **e** NK cells isolated from patients were treated with conditioned media (*CM*) from NK cells, MSCs, MSC/NK cell co-cultures (all from healthy controls), and cell-free medium each in the presence of neutralizing antibodies against CCL2 or the isotype control. Thereafter, all cells were stimulated with IL-12 and IL-18. The release of IFN-γ into the supernatant was determined 24 h later. **d** Bar graph showing mean ± SD of 4 replicates from one representative experiment. **e** Scatter plot showing individual values obtained from NK cells of six patients. Data were normalized to the values from NK cells treated with “medium” (*Med*) and stimulated in the presence of the isotype control (set as 100 %). *Horizontal lines* indicate the median with interquartile range. Statistical analysis was performed using the Mann–Whitney *U* test (**a**), one-way ANOVA followed by Bonferroni multiple comparison test (**b**, **d**) or Wilcoxon signed-rank test (**c**, **e**). ***p < 0.001, **p < 0.01, *p < 0.05. *MSC* mesenchymal stromal/stem cell, *NK* natural killer, *CCL2* C-C ligand 2, *IFN* interferon

Next, we asked whether MSCs exert their stimulatory capacity not only under physiological conditions but also under pathological conditions that are associated with suppressed NK cell function. We have evidence that NK cells from critically ill patients after major injury and surgery are impaired in function [14, 15]. In a small pilot study, we investigated the IFN-γ release from NK cells isolated from patients after severe injury and evaluated whether it was modulated by MSCs from healthy individuals. NK cells from critically ill patients and from healthy donors (for characteristics see Table 1) were therefore stimulated with IL-12 and IL-18. NK cells from patients released much less IFN-γ than NK cells from control subjects (Fig. 7a). The synthesis of IFN-γ was increased when the patients’ NK cells were cultured together with MSCs (Fig. 7b, c). The stimulatory effect of MSCs on these NK cells was partially inhibited by neutralization of CCL2/MCP-1 (Fig. 7b, c).

Moreover, the stimulatory effect of conditioned media from cells of healthy donors on NK cells from severely injured patients was evaluated. Conditioned medium from MSCs and from MSC/NK cell co-cultures of healthy donors increased the IL-12/IL-18-induced IFN-γ secretion from the patients’ NK cells in a CCL2/MCP-1-dependent manner (Fig. 7d). Overall, the stimulatory activity of conditioned media from MSCs and MSC/NK co-cultures on the patients’ NK cells was 2-fold and 3.3-fold, respectively (Fig. 7e). In the absence of IL-12 and IL-18, the patients’ NK cells did not release detectable levels of IFN-γ (data not shown). The CCL2/MCP-1-mediated activity of MSCs is thus not restricted to NK cells under homeostasis but also seems to be applicable to NK cells that are suppressed in function due to critical illness.

## Discussion

The majority of studies on the immunomodulatory activity of MSCs report the inhibitory effect of MSCs on diverse cells of the immune system. The key factors in human MSC-mediated immunoregulation are IDO, PGE_2_, and TGF-β [5]. There exist only few reports on proinflammatory properties of MSCs that deal with the attraction and stimulation of granulocytes or macrophages [16, 17]. We have previously identified another proinflammatory activity of MSCs because they increase the production of IL-12 and IL-18-induced IFN-γ from NK-92 cells through unknown mechanisms [11]. We show here that MSCs exerted their stimulatory activity also on human primary NK cells during cell–cell contact as well as through a soluble factor. In line with previous reports, the minor CD56^bright^ NK cell population was superior in IL-12/IL-18-induced IFN-γ production compared with the CD56^dim^ NK cell subtype [18]. Accordingly, the MSC-mediated stimulation of NK cells was more apparent in CD56^bright^ NK cells. Overall, we observed a broad range of the amount of CCL2 and IFN-γ that was released from MSCs and NK cells from different donors, respectively. A donor-dependent variation of the size of the CD56^bright^ NK cell population among total NK cells might contribute to the diverse IFN-γ production of the NK cells. The finding that conditioned medium from MSC/NK cell co-cultures was more potent in NK cell stimulation than conditioned medium from MSCs alone indicates that an immunostimulatory factor was released during the interaction of MSCs and NK cells.

Mechanistically, we identified chemokine receptor CCR2 and its ligand CCL2/MCP-1 as the major components in the MSC-mediated stimulation of NK cells. MSCs constitutively secrete CCL2/MCP-1 and other chemokines [19]. CCR2, the receptor for CCL2/MCP-1, is expressed on diverse immune cells, such as monocytes/macrophages, dendritic cells, T cells, and NK cells. CCL2/MCP-1 so far seemed to contribute to the immunosuppressive properties of MSCs. MSC-derived CCL2/MCP-1 attracts macrophages to tumors and thereby promotes tumor growth [20]. Likewise, murine T cells are recruited by MSC-derived CCL2/MCP-1 and subsequently are suppressed by NO that is released into the microenvironment by murine MSCs [21]. In addition to its chemotactic activity, MSC-derived CCL2/MCP-1 directly modulates cellular functions of target cells, such as the immunoglobulin production by plasma cells and the inhibition of Th17 T cells [22, 23].

In line with a previous study we observed that CD56^bright^ NK cells displayed a stronger expression of the CCR2 than CD56^dim^ NK cells [24]. The knowledge on the impact of CCL2/MCP-1 on NK cells is largely restricted to its chemotactic properties [24, 25]. We provide the novel finding that MSC-derived CCL2/MCP-1 directly acts on resting CD56^bright^ NK cells that subsequently respond to IL-12 and IL-18 with an increased production of IFN-γ. The weak expression of the CCR2 on CD56^dim^ NK cells might explain why MSCs do not promote but rather reduce the low production of IFN-γ from CD56^dim^ NK cells when examined immediately after stimulation [26]. The finding that blocking of CCL2/MCP-1 in conditioned media from MSCs/NK cell co-cultures was less effective than inhibition of the CCR2 on NK cells led to the assumption that CCL2/MCP-1 was not the only factor which triggered CCR2 on CD56^bright^ NK cells. Indeed, alternative ligands of CCR2—namely CCL7, CCL8, and CCL12—displayed similar stimulatory activity on CD56^bright^ NK cells and might also have been present in the conditioned media from MSCs.

It remains unclear how CCL2/MCP-1-mediated signaling interacts with the IL-12/IL-18-induced pathway in CD56^bright^ NK cells. Activation of suppressor of cytokine signaling 3 (SOCS3) and suppression of T-box expressed in T cells (T-bet) were identified as mechanistic targets of CCL2 secreted from decidual stromal cells on diverse leukocytes [19, 27]. However, transcription of the *ifng* gene requires the expression of T-bet and is suppressed by SOCS3 [28–30]. Therefore, we assume that signaling pathways distinct to activation of SOCS3 and suppression of T-bet are induced in CD56^bright^ NK cells after exposure to MSC-derived CCL2 in order to increase their IFN-γ response.

Several studies in the past have shown that MSCs require licensing by exogenously added IFN-γ and tumor necrosis factor alpha or by specific toll-like receptor ligands to acquire immunosuppressive effects [31–34]. In our study, the interaction of MSCs and NK cells occurred in the absence of exogenous proinflammatory or anti-inflammatory factors. Minute amounts of IFN-γ that were produced in the MSC/NK cell co-cultures were sufficient to further drive the release of CCL2/MCP-1 from MSCs, indicating that NK cells licensed MSCs in the neutral environment. On the contrary, the bidirectional interaction with MSCs primed NK cells in a CCR2-dependent manner for increased IFN-γ secretion upon exposure to IL-12 and IL-18. This immunostimulatory feedback loop between MSCs and NK cells represents a novel aspect in the plasticity of MSC function.

NK cells maintained their enhanced responsiveness to IL-12 and IL-18 even after detachment from MSCs and removal of MSC-derived factors. This is in contrast to the well-studied MSC-mediated suppression of T-cell responsiveness that requires a closer vicinity between MSCs and T cells as the immunosuppressive factors NO and IDO act only over a short distance [21, 35]. Transferred to a potential in-vivo situation, we speculate that, after priming by MSCs, CD56^bright^ NK cells may support inflammation both locally and distant to the site of MSC encounter; for example, upon infection that is associated with the release of IL-12 and IL-18 [36, 37]. It is important to note that MSC-derived CCL2/MCP-1 per se did not induce the release of IFN-γ from NK cells. Thus, it is unlikely that MSCs cause uncontrolled inflammation in the absence of, for example, an infectious insult. Moreover, considering the importance of NK cell-derived IFN-γ in T-helper cell polarization in lymphoid organs, such MSC-primed NK cells might favor the development of inflammatory T-helper cell type 1 responses [38]. Further studies are required to evaluate the existence and the consequences of MSC-mediated NK cell priming in vivo, especially under the view of the growing number of clinical studies based on the therapeutic application of MSCs.

The administration of anti-inflammatory MSCs in clinical trials has so far been aimed largely at treating hyperinflammatory diseases such as graft-versus-host disease and autoimmunity [39, 40]. Because of their inhibitory properties the use of MSCs in diseases associated with immunodeficiency has so far been regarded as contraindicative. Severe injuries or invasive surgical interventions cause a state of immunosuppression [12]. These patients suffer from nosocomial infections that may result in life-threatening septic shock. The underlying pathomechanisms of injury-induced immunosuppression are not yet fully understood and protective therapies are warranted. We have presented evidence that NK cells from severely injured patients are impaired in IFN-γ production upon exposure to *Staphylococcus aureus* [14]. In line with these data, we show here for the first time that NK cells from severely injured patients are suppressed in their capacity to secrete IFN-γ in response to IL-12/IL-18, the key cytokines in the defense against diverse pathogens. We observed that MSCs from healthy donors at least partially restored the suppressed IFN-γ synthesis of NK cells from such patients. Moreover, conditioned media from MSCs as well as from co-cultures of MSCs and NK cells from healthy donors clearly improved the IFN-γ release from NK cells of injured patients. Similar to our data obtained from NK cells from healthy donors, CCL2/MCP-1 mediated the stimulatory effect of MSCs and their conditioned media on the patients’ NK cells.

## Conclusions

In the neutral environment, trace amounts of NK cell-derived IFN-γ licensed MSCs for enhanced release of CCL2/MCP-1 that primed NK cells for increased secretion of IFN-γ upon exposure to IL-12 and IL-18. This novel positive feedback loop between MSCs and NK cells was not only observed for NK cells from healthy donors but also for NK cells from immunocompromised patients. Targeting MSCs or their soluble factors to the patients’ NK cells might improve the immune function after severe injury and, thereby, extend the indication of MSC therapy.

## Abbreviations

CCL, C-C chemokine ligand; CCR, C-C-chemokine receptor; ELISA, enzyme-linked immunosorbent assay; FCS, fetal calf serum; IDO, indoleamine 2,3-dioxygenase; IFN, interferon; IL, interleukin; MCP-1, monocyte-chemoattractant protein; MSC, mesenchymal stromal/stem cell; NK, natural killer; nmMSC, mesenchymal stromal/stem cell from nasal mucosa; PBMC, peripheral blood mononuclear cells; PGE, prostaglandin E; SOCS3, suppressor of cytokine signaling 3; T-bet, T-box expressed in T cells; TGF, transforming growth factor

## Additional files

Additional file 1: Figure S1. Expression of specific molecules on MSCs. MSCs were incubated with fluorochrome-labeled antibodies against CD45, CD31, CD34, CD73, CD105, CD29, and CD90, and with the respective isotype control antibodies. Representative histograms show the fluorescence of the isotype (*grey shaded area*) and the specific antibody (*black line*). *MSC* mesenchymal stromal/stem cell (PDF 48 kb) Additional file 2: Figure S2. CD56^dim^ NK cells do not respond to CCR2 ligands. NK cells were incubated in the absence (–) or presence of 0.5 ng/ml of recombinant human CCL2, CCL8, CCL7, and CCL12, or with conditioned medium (*CM*) from MSCs for 12 h. Thereafter, the cells were stimulated with IL-12 (1 ng/ml) and IL-18 (5 ng/ml). Dot plots of intracellular staining of IFN-γ in gated CD3^–^CD56^dim^ NK cells (gating strategy as shown in Fig. 1d). Data are representative for one out of five experiments. The threshold of positive staining for IFN-γ was set according to the isotype control (*iso*). Numbers indicate the percentage of IFN-γ-positive NK cells. *MSC* mesenchymal stromal/stem cell, *CCL* C-C ligand, *IFN* interferon. (PDF 36 kb)
