# Supplementary material for: Human mesenchymal stromal/stem cells acquire immunostimulatory capacity upon cross-talk with natural killer cells and might improve the NK cell function of immunocompromised patients
Source: Stem Cell Res Ther. 2016 Jul 7;7:88. doi: 10.1186/s13287-016-0353-9 (PMC4937587; doi:10.1186/s13287-016-0353-9)
Supplement: Additional file 1: Figure S1. — Expression of specific molecules on MSCs. MSCs were incubated with fluorochrome-labeled antibodies against CD45, CD31, CD34, CD73, CD105, CD29, and CD90, and with the respective isotype control antibodies. Representative histograms show the fluorescence of the isotype (grey shaded area) and the specific antibody (black line). MSC mesenchymal stromal/stem cell (PDF 48 kb) [file 13287_2016_353_MOESM1_ESM.pdf]

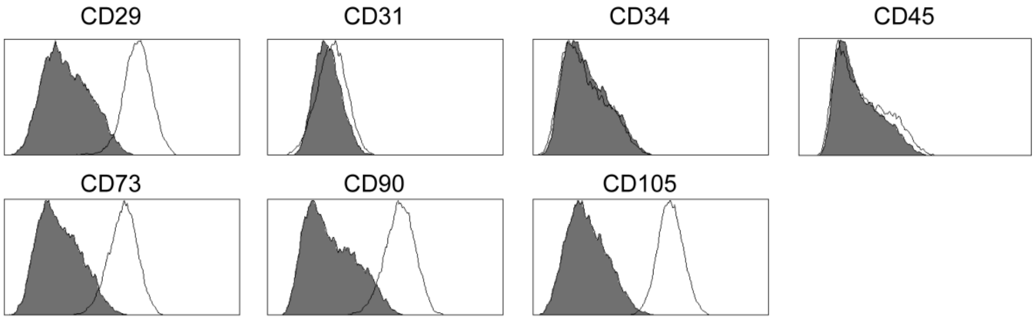

Additional file Figure 1

Expression of specific molecules on MSCs. MSCs were incubated with fluorochrome-labeled antibodies against CD45, CD31, CD34, CD73, CD105, CD29, CD90, and with the respective isotype control antibodies. Representative histograms shows the fluorescence of the isotype (grey shaded area) and the specific antibody (black line). MSCs, mesenchymal stromal/stem cells
