# Supplementary material for: Human mesenchymal stromal/stem cells acquire immunostimulatory capacity upon cross-talk with natural killer cells and might improve the NK cell function of immunocompromised patients
Source: Stem Cell Res Ther. 2016 Jul 7;7:88. doi: 10.1186/s13287-016-0353-9 (PMC4937587; doi:10.1186/s13287-016-0353-9)
Supplement: Additional file 2: Figure S2. — CD56dim NK cells do not respond to CCR2 ligands. NK cells were incubated in the absence (–) or presence of 0.5 ng/ml of recombinant human CCL2, CCL8, CCL7, and CCL12, or with conditioned medium (CM) from MSCs for 12 h. Thereafter, the cells were stimulated with IL-12 (1 ng/ml) and IL-18 (5 ng/ml). Dot plots of intracellular staining of IFN-γ in gated CD3–CD56dim NK cells (gating strategy as shown in Fig. 1d). Data are representative for one out of five experiments. The threshold of positive staining for IFN-γ was set according to the isotype control (iso). Numbers indicate the percentage of IFN-γ-positive NK cells. MSC mesenchymal stromal/stem cell, CCL C-C ligand, IFN interferon. (PDF 36 kb) [file 13287_2016_353_MOESM2_ESM.pdf]

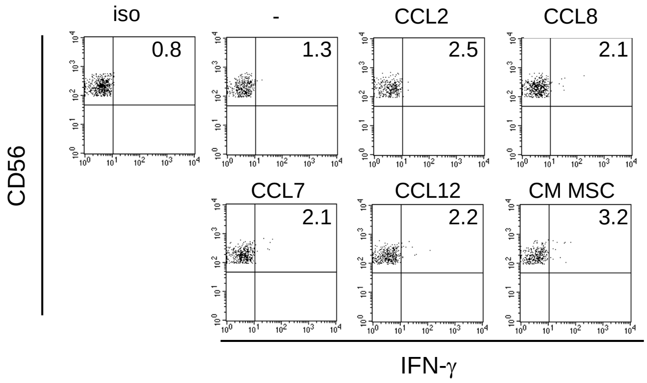

Additional file Figure 2

CD56^{dim} NK cells do not respond to CCR2 ligands. NK cells were incubated in the absence (-) or presence of 0.5 ng/ml of recombinant human CCL2, CCL8, CCL7, and CCL12, or with conditioned medium (CM) from MSCs for 12 h. Thereafter, the cells were stimulated with IL-12 (1 ng/ml) and IL-18 (5 ng/ml). Dot plots of intracellular staining of IFN-γ in gated CD3^{-}CD56^{dim} NK cell (gating strategy as shown in Fig. 1D). Data is representative for one out of five experiments. The threshold of positive staining for IFN-γ was set according to the isotype control (iso). Numbers indicate the percentage of IFN-γ-positive NK cells. MSCs, mesenchymal stromal/stem cells; NK cell, natural killer cell; CCL2, C-C ligand 2; CCR, C-C chemokine receptor; IFN, Interferon
